# Resolution of refractory chylothorax with a combination of talc pleurodesis and CPAP

**DOI:** 10.1002/rcr2.624

**Published:** 2020-07-15

**Authors:** Boon Hau Ng, Nik Nuratiqah Nik Abeed, Mohamed Faisal Abdul Hamid, Chun Ian Soo, Hsueh Jing Low, Andrea Yu‐Lin Ban

**Affiliations:** ^1^ Pulmonology Unit, Department of Medicine, Faculty of Medicine Universiti Kebangsaan Malaysia Medical Centre Kuala Lumpur Malaysia; ^2^ Department of Anaesthesiology and Critical Care, Faculty of Medicine Universiti Kebangsaan Malaysia Medical Centre Kuala Lumpur Malaysia

**Keywords:** Chylothorax, CPAP, refractory, talc pleurodesis

## Abstract

Chylothorax is an uncommon cause of pleural effusion. Recurrent chylous effusions are often resistant to conservative treatment and many need surgical intervention. We report a 69‐year‐old woman with refractory idiopathic chylothorax resistant to medium‐chain triglyceride diet and intermittent thoracentesis. Lymphangiography and lymphoscintigraphy failed to identify the site of leakage. We initiated continuous positive airway pressure (CPAP) 12 h before and 48 hours after talc pleurodesis. Chest drain was removed at day 3 and she was discharged at day 5. To our knowledge, this is the first case of successful resolution of idiopathic refractory chylothorax with CPAP ventilation used in tandem with talc pleurodesis.

## Graphical abstract

We describe a 69‐year‐old woman with underlying diffuse large B‐cell lymphoma of the colon 16 years ago in remission, who presented with a new problem of refractory chylothorax. It was successfully treated with continuous positive airway pressure (CPAP) ventilation in tandem with talc pleurodesis.

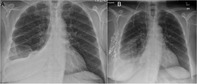

## Introduction

Chylothorax is a rare and debilitating disease. It can be congenital or result from obstruction or neoplasm. The treatment varies from conservative dietary modifications, treatment of the underlying cause, pleurodesis, or surgical ligation of the thoracic duct. Continuous drainage of chyle into the pleural cavity causes serious nutritional deficiency and respiratory complications. Refractory chylothorax is often difficult to treat, and its diagnosis and management often presents a significant problem to clinicians.

## Case Report

**Figure 1:**
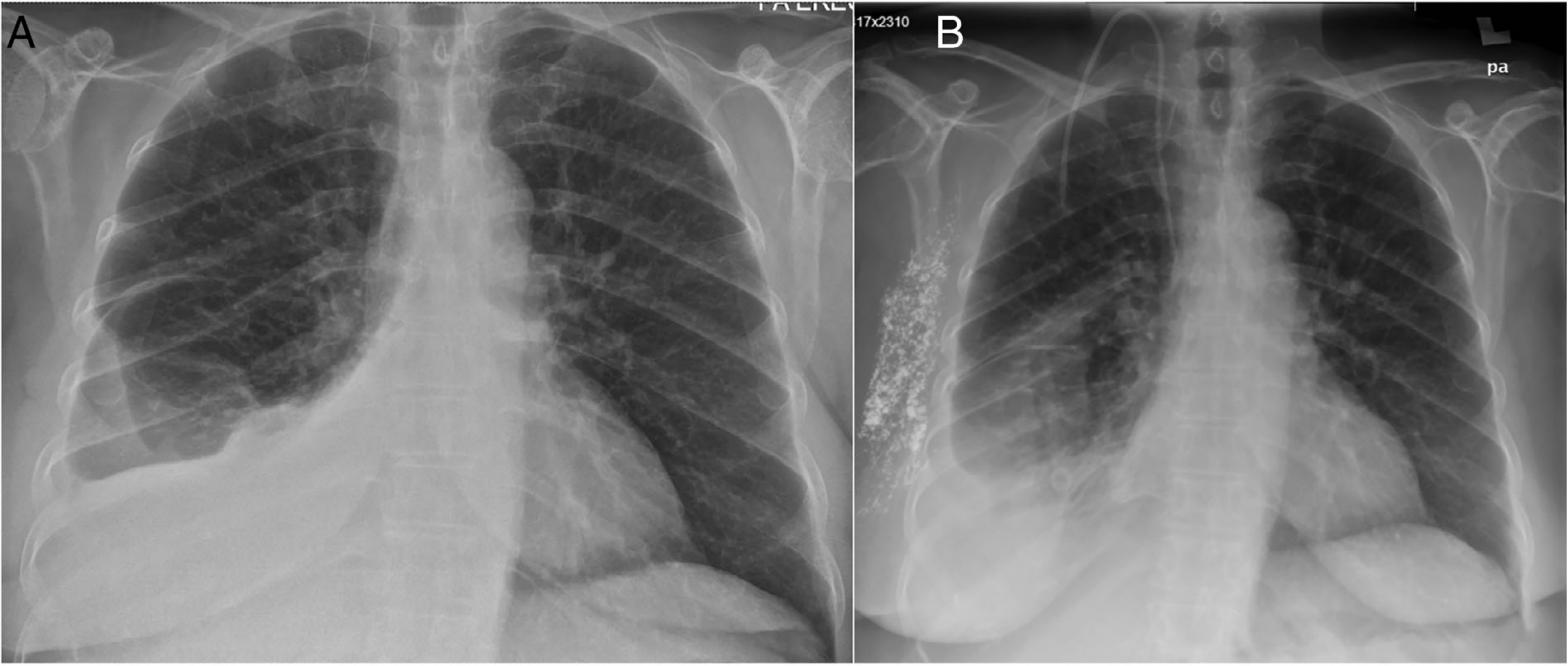
(A) Chest radiograph on admission showing moderate right pleural effusion. (B) Chest radiograph with resolution of right chylothorax. Also seen is the contrast medium from lymphangiogram and a central line in situ.

A 69‐year‐old woman initially presented to a gastroenterologist with a vague history of abdominal discomfort and constipation. She was found to have an incidental right‐sided pleural effusion on chest radiograph (Fig. 1A). Clinical examination was in keeping with a pleural effusion with a normal abdominal system. She had a history of diffuse large B‐cell lymphoma (DLBCL) of the colon 16 years ago and was in complete remission. Thoracentesis confirmed right chylothorax with pleural triglyceride of 2.13 mmol/L. Pleural fluid for tuberculous direct smear, cultures, and polymerase chain reaction was negative. Pleural fluid cell block showed histiocytes (CD68+) with scattered reactive mesothelial cells and normal reactive T (CD3+) and B (CD20+) lymphocytes. A computed tomography (CT) scan revealed no enlarged lymph nodes.

She had a normal colonoscopy examination and colonic biopsy. Two separate lymphangiography sessions, one via the right inguinal and the other via the right axillary (Fig. 1B), failed to show significant deposits in the pleural cavity. We then subjected her to a lymphoscintigraphy which showed abnormal tracer accumulation in the lateral aspect of the right third rib. She had a negative whole‐body positron emission tomography scan.

Despite total parenteral nutrition (TPN) of medium‐chain triglyceride (MCT) with a low‐fat diet, she required therapeutic thoracentesis of 1.5 L of chyle every two weeks. There was persistent drainage of the chylothorax of up to 300 mL daily for a further four weeks. We then consulted the cardiothoracic team for a possible thoracic duct ligation. A video‐assisted thoracoscopy with methylene blue injection into the right axillary lymph nodes failed to identify the site of leakage.

We then performed talc pleurodesis. We aspirated the chyle completely through a 32‐F chest drain and initiated her on 12 h of continuous positive airways pressure (CPAP) at 10 cm H_2_O with fraction of inspired oxygen (FiO_2_) of 40%. We confirmed the absence of residual effusion with a bedside thoracic sonography the following day and proceeded with talc slurry pleurodesis using 5 g of talcum followed by a further 48 h of CPAP at the same pressure. We removed the chest drain at day 3, after confirming the resolution of the effusion by a bedside thoracic sonography. She was discharged at day 5. There was no evidence of reaccumulating effusion at week 2, week 6, and three months.

## Discussion

Chylothorax is an abnormal collection of chyle in the pleural cavity which results from an anatomical disruption of the thoracic duct or one of its main tributaries. The thoracic duct drains the lymphatics from lumbar, lower extremities, intestine, and liver. When the duct is injured, the chyle leaks into the mediastinum and pleural space. The causes of chylothorax can be divided into surgical and medical. The most common one is the medical cause lymphoma. The workout from our patient did not suggest a relapse of lymphoma.

The diagnosis of chylothorax is confirmed when the pleural fluid triglyceride concentration is >110 mg/dL with cholesterol level <200 mg/dL or the presence of chylomicron by lipoprotein electrophoresis. Persistent leakage can result in nutritional deterioration and an immune deficiency state. Conservative management includes MCT diet, TPN, and somatostatin analogue octreotide. Both lymphoscintigraphy and lymphangiography are lymphatic imaging studies which can play a therapeutic role [1]. In our patient, both these procedures failed to detect the site of leak and had no therapeutic benefit.

Standard treatment for refractory idiopathic chylothorax has not been established. Pleurodesis is well validated in malignant pleural effusion with reported success rate up to 76% [2]. There is less evidence to support its use in non‐malignant effusions like chylothorax. Talc pleurodesis at 4–8 g in lymphoma‐related chylothorax showed a 100% success rate in a small case series [3].

The success of pleurodesis requires re‐expansion of the lung and apposition of the visceral and parietal pleura. CPAP has been shown to increase the intrapleural pressure which promotes drainage and reabsorption of pleural fluid leading to better lung expansion [4]. CPAP of 10 cm H_2_O and 200 mg of minocycline were shown to be effective in post oesophagectomy chylothorax with drainage ceasing at 30 h post combined treatment [5]. We used a similar CPAP with 5 g of talc and drainage ceased earlier at 24 h. We were able to remove the tube at 72 h post procedure.

A combination of talc pleurodesis and CPAP for adequate lung expansion appears effective, minimally invasive, and well tolerated. Our method may be a treatment option for patients with idiopathic refractory chylothorax not responding to conservative treatment and in whom surgery is not an option due to failure to locate the site of leakage. Further studies are needed to validate the use of CPAP and talc pleurodesis in the management of refractory chylothorax.

### Disclosure Statement

Appropriate written informed consent was obtained for publication of this case report and accompanying images.
